# Clinical Significance of the Identification of an Anti-M (IgG) Alloantibody in Pregnancy

**DOI:** 10.7759/cureus.116240

**Published:** 2026-09-14

**Authors:** Alla Iashchuk, Ricardo Sardo

**Affiliations:** 1 Immuno-Hemotherapy, Unidade Local de Saúde de Trás-os-Montes e Alto Douro, Vila Real, PRT; 2 Immuno-Hemotherapy, Unidade Local de Saúde do Alto Minho, Viana do Castelo, PRT

**Keywords:** alloimmunization, anti-m antibody, fetal anemia, hemolytic disease of the fetus and newborn, igg alloantibody, immunohematology, mns blood group system, pregnancy

## Abstract

Anti-M is usually a naturally occurring, cold-reactive immunoglobulin M (IgM) antibody and is commonly regarded as clinically insignificant. An immunoglobulin G (IgG) component, however, can cross the placenta and has rarely been associated with hemolytic disease of the fetus and newborn (HDFN), including hyporegenerative fetal or neonatal anemia. A 42-year-old woman (G4P2A2) undergoing preconception evaluation had a positive indirect antiglobulin test (IAT) with initially inconclusive antibody identification. Reference immunohematology testing identified anti-M reactivity at 4°C. After dithiothreitol (DTT) treatment, an immunoglobulin G component was confirmed at a titer of 1:2. The direct antiglobulin test (DAT) remained negative, and there was no evidence of maternal hemolysis. Her partner was M antigen positive, indicating that a future fetus could inherit the target antigen. This case shows that detailed characterization of an apparently cold-reactive anti-M antibody can materially change preconception counseling and pregnancy planning. Low titer alone should not replace consideration of antibody class, thermal amplitude, obstetric history, paternal or fetal antigen status, and coordinated follow-up by transfusion medicine and maternal-fetal medicine teams.

## Introduction

Maternal red cell alloimmunization occurs when maternal antibodies are directed against red cell antigens inherited by the fetus. Immunoglobulin G (IgG) antibodies can cross the placenta and cause hemolytic disease of the fetus and newborn (HDFN), whereas immunoglobulin M (IgM) antibodies generally do not cross the placenta. Contemporary guidance therefore emphasizes accurate antibody identification, assessment of its clinical significance, and determination of whether the fetus carries the corresponding antigen [1,2].

The M and N antigens belong to the MNS blood group system and are carried on glycophorin A, a protein on the red cell membrane. Anti-M antibodies are frequently naturally occurring and represent a relatively common specificity among detected red cell antibodies. In a nationwide population study of pregnancies with positive antibody screening, anti-M accounted for 19.4% of identified red cell antibodies, making it one of the most frequently detected specificities [3]. These antibodies react most strongly at temperatures below 37°C, often display dosage, and are usually predominantly IgM. Consequently, in most pregnancies, anti-M is not associated with clinically important fetal disease. Nevertheless, some anti-M antibodies contain an IgG component capable of crossing the placenta, and rare reports describe fetal anemia, hydrops, intrauterine death, neonatal hyperbilirubinemia, and prolonged hyporegenerative anemia [4-10]. The latter may result from antibody-mediated destruction not only of mature red cells but also of erythroid precursors. Therefore, the neonatal direct antiglobulin test (DAT), which detects antibodies bound to red cells, can be weak or negative despite clinically important disease [5,8,9].

Clinically significant IgG anti-M alloimmunization is rare, and its heterogeneous behavior makes risk stratification difficult. Importantly, there is no universally accepted critical anti-M titer that reliably predicts fetal disease. Large institutional experience suggests that severe disease is uncommon, while recent severe cases demonstrate that adverse outcomes may occur even when titers are not strikingly high [4,6,10-13]. For this reason, individualized risk assessment is necessary. Maternal titer, thermal amplitude, IgG status, prior obstetric history, paternal or fetal antigen status, and evidence of fetal anemia should be interpreted together rather than through a single anti-M threshold.

We report the preconception identification of a low-titer IgG anti-M antibody in a woman with prior miscarriages and an M-positive partner. The case illustrates how reference laboratory characterization of an antibody initially considered cold-reactive and benign altered counseling and the proposed surveillance strategy for a future pregnancy.

## Case presentation

A 42-year-old woman (G4P2A2) with two uncomplicated term pregnancies and two miscarriages with different partners, was attempting to conceive. She had no known history of blood transfusion. Initial testing showed blood group B, RhD positive, an extended phenotype reported as CcDee K+, a negative DAT, and a positive indirect antiglobulin test (IAT). The initial antibody identification panel was inconclusive.

The sample was subsequently evaluated at the Portuguese Institute of Blood and Transplantation in Porto. Anti-M was identified, with reactivity restricted to heterozygous M+N+ reagent cells at 4°C. This pattern was initially considered compatible with a predominantly cold-reactive IgM antibody. On reassessment after dithiothreitol (DTT) treatment, which disrupts pentameric IgM, residual anti-M reactivity confirmed an IgG component. The IgG anti-M titer was 1:2 when tested against M+N+ cells.

The DAT remained negative throughout the investigation, and no evidence of maternal hemolysis was reported. Partner testing showed blood group A, RhD positive, with the phenotype M+N+S+s+. The partner's M-positive status means that a future fetus may inherit the M antigen, although paternal heterozygosity does not establish fetal antigen status. All available immunohematologic investigations are summarized in Table 1. No additional hematology or chemistry investigations were available in the supplied clinical record.

**Table 1:** Available maternal and partner immunohematologic investigations Phenotypes and antibody titers do not have conventional numeric reference intervals; the expected result or clinical interpretation is therefore provided. DAT: direct antiglobulin test, DTT: dithiothreitol, IAT: indirect antiglobulin test

Investigation	Result	Reference/clinical meaning
Maternal ABO group	B	Phenotypic result; no reference interval
Maternal RhD type	Positive	RhD antigen detected
Maternal extended phenotype	CcDee K+	Reported red cell phenotype; no reference interval
DAT	Negative	Expected: negative; no detectable in vivo red cell coating
IAT	Positive	Expected screen: negative; unexpected serum antibody detected
Initial antibody identification	Inconclusive	Expected: no unexpected alloantibody; reference testing required
Anti-M testing at 4°C	Reactive with M+N+ cells	Expected: nonreactive; cold reactivity with dosage pattern
Anti-M after DTT treatment	Residual reactivity; IgG confirmed	Expected: nonreactive after IgM disruption
IgG anti-M titer	1:2 against M+N+ cells	Low titer; no universally accepted critical anti-M titer
Maternal hemolysis	Not detected or reported	Expected: absent
Partner ABO group	A	Phenotypic result; no reference interval
Partner RhD type	Positive	RhD antigen detected
Partner MNS phenotype	M+N+S+s+	M-positive; compatible with MN heterozygosity
Fetal M-antigen status	Not tested (preconception)	Not applicable; a future fetus may be M-positive

## Discussion

This case is clinically relevant because an antibody initially characterized by cold-only reactivity was shown, after DTT treatment, to contain an IgG component. DTT disrupts the disulfide bonds of IgM and can help distinguish an IgM-dominant anti-M from persistent IgG activity. The result does not by itself predict fetal disease, but it changes the antibody from a routine cold-reactive finding into one that merits pregnancy-specific risk assessment.

The overall probability of severe anti-M-mediated HDFN remains low. In a previously published single-institution series comprising 195 pregnancies, most titers were low and no case of HDFN was identified [4]. These findings provide useful contextual evidence regarding the generally low risk of severe disease but are not directly comparable with the present patient. This reassuring experience must be balanced against the published spectrum of disease. A recent systematic review collated cases ranging from asymptomatic antigen-positive neonates to fetal loss and severe anemia requiring antenatal intervention [6]. Published cases are susceptible to referral and publication bias, so their severity cannot be used to estimate an individual patient's absolute risk.

The present titer of 1:2 is reassuring but not definitive. Severe disease has generally been associated with higher or rising titers and adverse obstetric history, yet low titers have occasionally preceded important disease [6,10,12]. Anti-M titration is method-dependent, dosage may affect reactivity, and titers correlate imperfectly with disease severity. Accordingly, a single anti-M titer should be interpreted as a baseline for comparison in the same laboratory rather than as a stand-alone prognostic test. The patient's two miscarriages cannot be attributed to anti-M from the available data, particularly because they occurred with different partners and no fetal antigen status or evidence of anemia was documented.

Paternal phenotyping refines but does not resolve fetal risk. The partner is M+N+, consistent with MN heterozygosity, so a future fetus may or may not inherit M. If the fetus is M-negative, maternal anti-M cannot cause HDFN. Unlike RhD, RhC/c, RhE, K, and Fya, noninvasive cell-free fetal DNA assays for M are not universally validated or routinely available [1,14]. Local availability should therefore be confirmed with a reference laboratory. If noninvasive testing is unavailable and pregnancy management would change materially, specialist counseling is needed before considering invasive genotyping.

A pragmatic plan begins with early antenatal confirmation of antibody specificity, IgG reactivity at 37°C or the antiglobulin phase, and a baseline titer performed by a consistent method. Repeat testing should be individualized according to the reference laboratory's protocol, titer trend, obstetric history, and fetal antigen status [1,4,14]. The literature does not support a universal anti-M-specific critical titer, and applying thresholds developed for anti-D or other antibodies without qualification may be misleading. Early consultation with transfusion medicine and maternal-fetal medicine is appropriate if the IgG component persists, the titer rises, fetal antigen status is positive or unknown, or the prior pregnancy history raises concern.

When a fetus is considered at meaningful risk, middle cerebral artery peak systolic velocity (MCA-PSV) Doppler is the preferred noninvasive test for fetal anemia. An MCA-PSV greater than 1.5 multiples of the median or hydrops should prompt referral to a center capable of fetal blood sampling and intrauterine transfusion [15]. Anti-M case reports document successful outcomes after serial intrauterine transfusions, while exceptional early severe disease has also been treated with immunomodulatory strategies [10-12]. These interventions are not justified by the current preconception titer alone; they become relevant only if a future pregnancy demonstrates an antigen-positive or antigen-unknown fetus with evidence of clinically significant alloimmunization or anemia.

Neonatal planning is also important because anti-M-associated anemia may be hyporegenerative and delayed. DAT negativity does not exclude disease, as shown in reports of prolonged anemia and reticulocytopenia [5,8,9]. If a future infant is M-positive, cord or early neonatal testing should include hemoglobin or hematocrit, bilirubin, reticulocyte count, DAT, and antigen typing, followed by clinical and laboratory reassessment when indicated. The transfusion service should be informed in advance because M-negative, crossmatch-compatible red cells may be required for the mother, fetus, or neonate.

This report is limited by its preconception timing and the narrow clinical dataset supplied. No pregnancy outcome, fetal genotype, fetal Doppler assessment, neonatal evaluation, or complete blood count and hemolysis panel was available. The significance of the two miscarriages therefore remains uncertain. Its strength is the stepwise reference laboratory investigation, including thermal-phase assessment, recognition of dosage, DTT testing, and partner phenotyping. These findings support a proportionate surveillance plan without overstating the risk associated with a low baseline titer.

## Conclusions

A low-titer anti-M antibody should not automatically be dismissed when an IgG component is confirmed. In this preconception case, DTT testing confirmed an IgG component despite a low anti-M titer of 1:2, while partner phenotyping demonstrated an M-positive status and therefore a biologically plausible risk to an M-positive future fetus. At the same time, the low maternal titer and absence of maternal hemolysis remained reassuring features.

Management should be individualized and coordinated among obstetric, transfusion medicine, and fetal medicine teams. Early reassessment in pregnancy, consistent laboratory methods, consideration of fetal antigen status, and escalation to fetal anemia surveillance only when clinically indicated can balance vigilance with avoidance of unnecessary intervention. Neonatal follow-up should account for the possibility of delayed or hyporegenerative anemia even when the DAT is negative.
